# Self-reported Perceptions of Weight and Eating Behavior of School Children in Sunderland, England

**DOI:** 10.3389/fpubh.2017.00017

**Published:** 2017-02-14

**Authors:** Alison McInnes, David Blackwell

**Affiliations:** ^1^Faculty of Health and Life Sciences, Northumbria University, Newcastle upon Tyne, UK; ^2^Faculty of Applied Sciences, The David Goldman Informatics Centre, The University of Sunderland, Sunderland, UK

**Keywords:** obesity, England, Sunderland, school children, eating patterns, eating behaviours

## Abstract

The main aim of this study was to determine the self-reported perceptions of weight and eating behaviors of school-age children in Sunderland in the North East of England. The results presented are derived from data collected by a Health-Related Behaviour Survey developed by Schools and Students Health Education Unit at Exeter University, and this study is based on analysis of the data set collected for Sunderland. A total of 12,213 pupils from nine secondary schools completed the questionnaire biennially from 1996 to 2012. The sample included 12 and 13 year olds and 14 and 15 year olds. Various health and social issues related to perceptions of weight and eating behaviors were determined. Trends related to these issues were identified according to age and gender of respondents, and differences between the groups were highlighted. From the analysis, some interesting findings relating to eating patterns and weight perception amongst young people were ascertained. Females of both age groups reported a greater desire to lose weight than their male counterparts. The percentage of school children who reported having breakfast at home has increased progressively, as have those having lunch at school. The percentage of school children purchasing lunch from takeaway outlets has dramatically decreased. This is pleasing since health policy of limiting take out provision is high on government agenda and these trends can be used by policy makers to focus on continuing to improve school meals. The findings partly support other national data but also contradict the widely held beliefs around food and obesity in the North East of England.

## Introduction

Obesity levels among children and adults are in the main rising fast in the United Kingdom (UK) (1–3). Obesity in the UK poses a huge challenge in terms of individual and public health, societal impact, and financial burden (4–6). Public Health England (7), state that 19% of children in year 6 (10–11 years old) are classed as obese. There is a close association between obesity in children and socioeconomic deprivation (8, 9). The Health and Social Care Information Centre in 2010 indicated that the prevalence of obesity among 15 year olds was 16% in boys and 15% for girls. As a result, tackling childhood obesity has been targeted as a government priority in England and consequently a variety of different local and national initiatives have been established. In 2005, the nutritional content of children’s school lunches was criticized, leading to new standards in schools in 2008 (10).

Local authorities have been urged to cut obesity levels in children and have been called upon to use planning restrictions to limit the number of fast food outlets, particularly near schools (7). This is particularly pertinent to Sunderland where there are a high percentage of fast food outlets close to schools (11). It has been estimated that up to one-third of North East children aged 10 and 11 years are officially classed as overweight and obese (12, 13). The legacy of the industrial heritage and higher than average deprivation levels has been blamed for this increase (14).

There are approximately 68,300 children in Sunderland, and these have been labeled as the “most overweight in the country” (15). Across Sunderland, 21.1% of year 6 children are overweight, higher than the national average of 18.9%. Furthermore, 45% of Sunderland residents live in areas rated as being within the top 20% of the “most disadvantaged areas” in England. Obesity has an increased prevalence in economically and socially deprived areas including the North East of England, in the UK (16). This is a recent phenomenon as historically deprived areas tended to see higher levels of children who were undernourished and hence underweight. However, is now appears that malnutrition is manifested as obesity in certain instances. Brunt et al. (17) illustrate that, between 1995 and 2005, this situation has now been reversed. They found the gap between obesity levels in the most deprived areas, in cities such as Sunderland, had overtaken those in the least deprived areas. Moreover, the Childhood Measurement Program (18) demonstrated Sunderland has some of the highest levels of overweight children in the UK, with 27.8% of reception-aged children (5–6 years old) being overweight or obese and this rises to 38.4% for year 6 pupils (10–11 years old). In the UK, almost 20% of children are obese by the time they leave primary school at 11 (19).

Two theories have been posited about problematic eating behavior of Sunderland school children. The first theory is that a large percentage of pupils arrive at school hungry (20–22). The NASUWT’s (22) findings show that almost three-quarters (74%) of teachers have observed pupils coming to school hungry, with 80% highlighting that school children are lacking in energy and concentration because they are eating poorly. With regard to undernourishment, the Government has agreed to allocate money to help schools in the most deprived areas to establish breakfast clubs and to extend free school meal entitlement, to ensure that the children of the so-called “working poor” do not go hungry (19). The second theory is that female school children skip meals to lose weight. This observation is based upon contemporary research widely reported in the local and national media (23, 24).

Conversely, a downward trend in obesity has been reported in the North East of England (5, 25). Overall the percentage of overweight 10–11 year olds has declined in six out of the North East’s seven Primary Care Trusts catchment areas (26). Furthermore, there has been a reported decrease in obesity levels for children in Sunderland (27).

This study by eliciting the subjects’ own perceptions facilitates comparison between these and the widely held views of the public, regarding various behaviors of school children. These include problematic drug and alcohol use, smoking, exercise, and eating behaviors. By providing a different perspective on the topic, this study will expand the overall knowledge base concerning eating behaviors of school children in a typical deprived postindustrial provincial city.

## Materials and Methods

The focus of this study is on the eating behaviors of school-age children in Sunderland. The data are derived from a survey carried out in Sunderland using the Health-Related Behavior Questionnaire (HRBQ) developed by the Schools and Students Health Education Unit (SHEU), and this study is based on analysis of the data set collected for Sunderland. The questionnaire was administered by SHEU to a total of 12,213 Sunderland school-aged children over a period of 16 years and using nine surveys. Two age groups: year 8 (12–13 year olds) and year 10 (14–15 year olds) were sampled. When the survey was administered, the school children’s ages fell within these age ranges. Since this is a cross-sectional survey, each time it takes place a sample from these two age ranges is taken. The samples included 1,476 pupils in 1996, 1,426 in 1998, 1,459 in 2000, 1,232 in 2002, 1,059 in 2004, 1,356 in 2006, 892 in 2008, 2,273 in 2010, and 1,040 in 2012. Nine secondary schools, used each time, were sampled biennially. These schools were geographically spread across the city. The catchment areas for each of these comprised of mixed socioeconomic groups, which have remained relatively stable over the 16 years of the study.

The questionnaire was administered by school teachers who were recruited and trained by staff from SHEU. With regard to ethical approval, the authors did not carry out the data collection and had no direct or indirect contact with the subjects. The data used for this study is not openly available and permission was received from Elouise Robinson, People Services Directorate, www.sunderland.gov.uk to use it. The authors analyzed the data set that was given to them. This proven methodology has been used by SHEU for 20 years and has been accepted by the Education and Public Health organizations in all of the authorities where the research has taken place. Furthermore, results have been analyzed, reported, and published by numerous authors over the period. Any issues around the methodology are within the remit of SHEU.

The survey methodology is through school based questionnaires originated within Exeter University, which are administered on a biennial basis. The HRBQ was first developed in 1977 as an outcome of research within the Department of Community Medicine at Nottingham University. Since then it has been used in thousands of school health surveys in secondary schools in the UK and also overseas. Across a period of more than 30 years, the methodology and content has been developed by SHEU in close consultation with schools throughout the UK. Each year the questionnaire has been further developed and refined in response to schools and partner agency feedback. The survey is under continual review to ensure that the questions are securing robust and reliable data. All participating schools were supported by SHEU in the collection of reliable data through pre-questionnaire half day seminar briefings, provision of a guide for conducting the questionnaire (which includes information on collecting good data, a supervisor’s guide and notes, and a telephone/e-mail advice service where schools could raise queries regarding any aspect of the survey).

The survey data produce a detailed profile of young people’s life at home, at school/college and with their friends. The information is then provided to health authorities to inform health needs assessments and health care planning and by schools/colleges to promote health education programmes, as well as in class work across the curriculum. To date, SHEU has supported thousands of health-related behavior surveys involving over a million young people. The data are anonymous and so no individual participant’s responses are attributable.

In this study, the biennial survey data provided by SHEU to Sunderland Health Authority on the topic area of eating habits from 1994 to 2014 are analyzed. Each survey is cross-sectional, and therefore, although this study covers a large time spam, it is not longitudinal in nature. However, it does provide information about the self-reported health behavior of school children of both genders in year 8 (12–13 years of age) and year 10 (14–15 years of age) of secondary schools within Sunderland for various topics every 2 years of the survey period. A standardized methodology was prescribed by SHEU with guidance and scrutiny undertaken by the unit’s staff. Full guidance notes for teachers were provided and training was given. This standardized procedure was consistent over time. All validation procedures were completed by SHEU, since they devised and administered this survey. All protocols are available on SHEU’s website www.sheu.org.uk (28). As this research was carried out with school children, it was designed in compliance with the Helsinki Declaration (http://www.wma.net/en/30publications/10policies/b3/index.html). Informed consent for participation in the study was obtained from a parent or guardian, in accordance with the Declaration of Helsinki. Sunderland Local Authority and the schools involved facilitated this process.

The methodology allows collection of data from a relatively large sample over an extended period of time; however, this is not a longitudinal study since the same individuals are not followed over time. It is a time related cross-sectional study, which permits the determination of trends within a field in which large studies are relatively uncommon. The SHEU questionnaire includes questions on eating behavior amongst other health-related topics. The reported behaviors of different ages and genders can be elicited and comparisons can be made and trends identified. From this, various health and social issues related to perceptions on weight, eating behavior, and food choices were identified. These issues have implications for the provision of services and long-term health needs of young people.

## Results

### Perceptions of Weight

**Figure 1:**
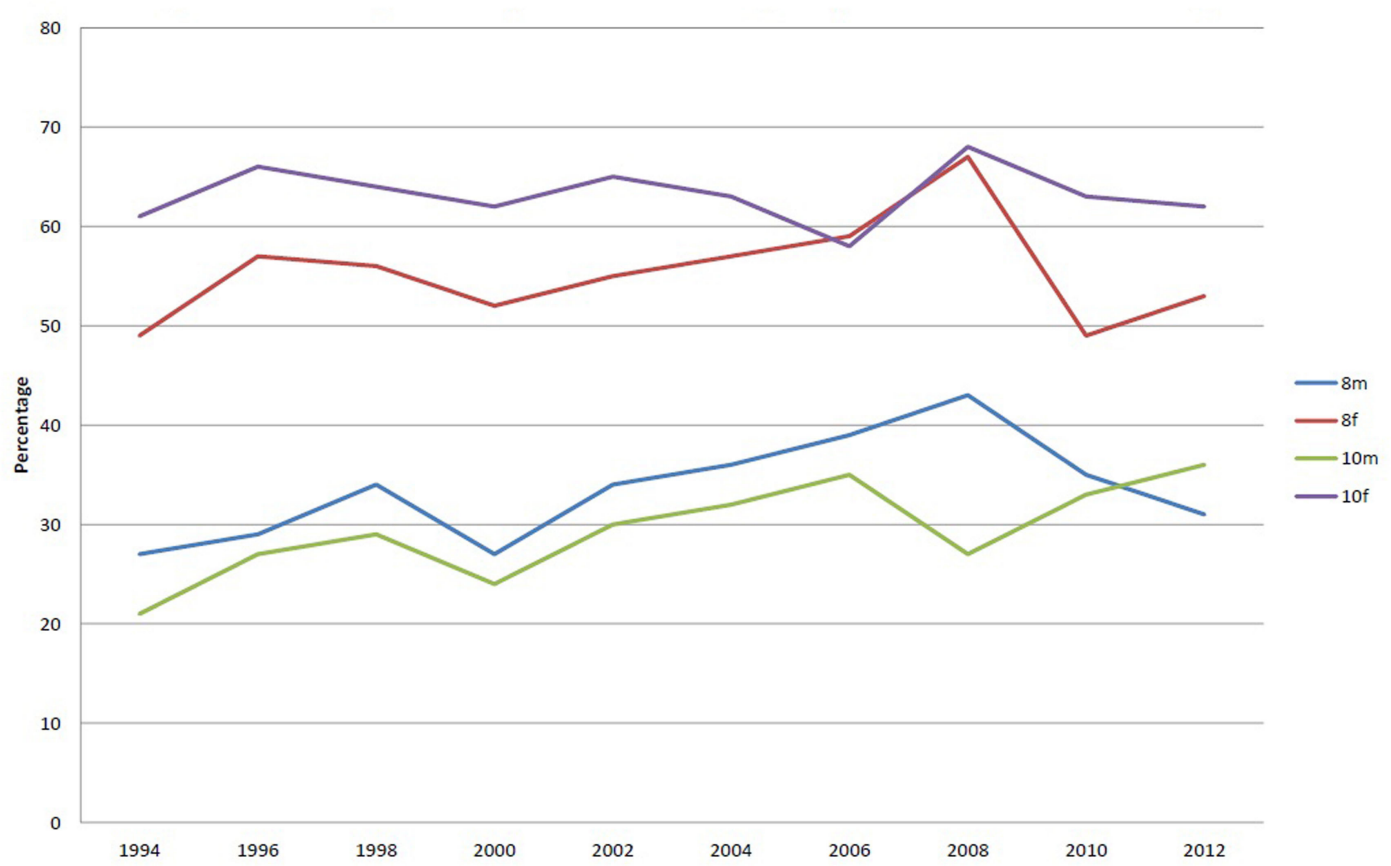
**Percentages of respondents stating they would like to lose weight**.

Figure 1 shows the percentage of respondents who wish to lose weight. It can be seen that females of both age groups report a greater desire to lose weight than their male counterparts. There is a striking difference between the female and male responses on this with consistently over 50% of females wishing to lose weight. Year 10, females are the group reporting the largest percentage wishing to lose weight. The percentage has remained consistently over 60% over the survey period, reaching a peak of 68% in 2006.

The percentage of year 8 females reporting they wish to lose weight increased progressively from 1994 to 2008 equaling the peak of year 10 females but has fallen since then to its original level. The percentage of males reporting a wish to lose weight has been consistently lower than females but has progressively increased over the study period.

A higher percentage of males in year 8, in comparison with those in year 10, reported wishing to lose weight. This peaked in 2008 at 42% but has fallen thereafter and has been overtaken by year 10 males in 2010 and 2012. These data show that over the study period the percentage of females wishing to lose weight is greater than those who report to be happy with their weight. This is exactly the opposite of the responses by males.

### Eating Behavior

**Figure 2:**
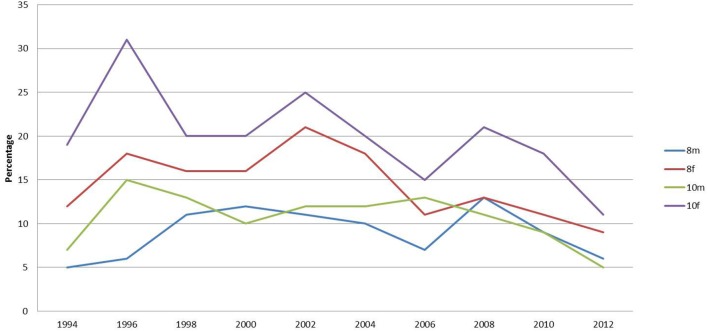
**Percentage of respondents reporting that they had nothing to eat or drink before school**.

The percentages reporting that they had nothing to eat or drink before coming to school has dropped most dramatically for year 10 females (Figure 2). Females were more likely than males to have nothing to eat before school. The percentage of this group reporting eating nothing before school was 32% in 1996 but dropped progressively to 12% in 2012. The percentage of year 8 females rose from 1994 to a peak of 22% but has fallen to 8% in 2012.

### Breakfast Eating Habits

**Figure 3:**
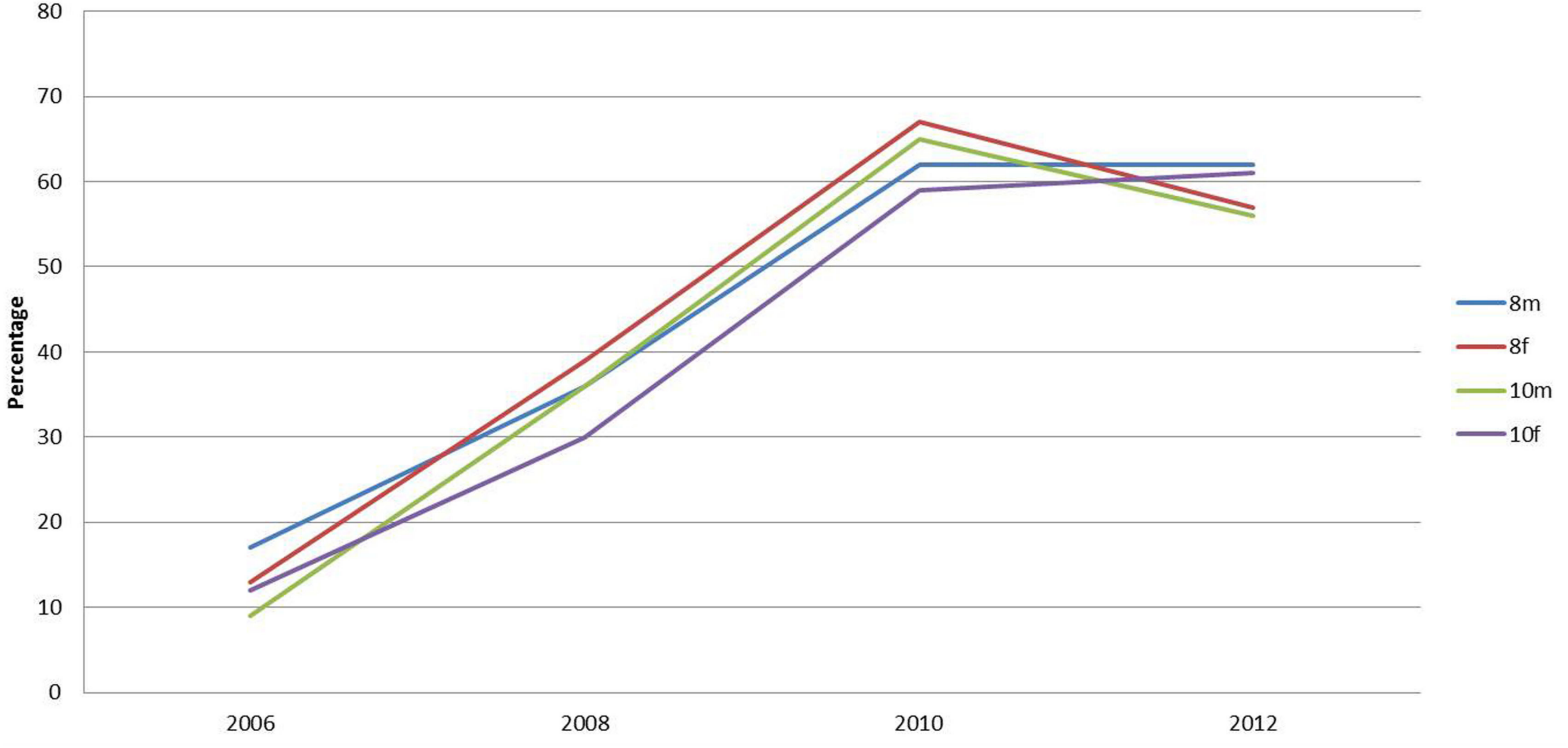
**Percentage of respondents stating they ate breakfast at home**.

From 2006, the survey has enquired about breakfast eating habits (Figure 3). The percentage of school children who reported having breakfast at home has increased progressively over the period (Figure 3). Of those eating breakfast, the traditional cooked breakfast has remained at a consistently low percentage, between 3 and 7%, over the period. The percentage of children eating cereals for breakfast has remained constant at a high level throughout (between 45 and 60%). Since 2000, when the question was introduced, the percentage of school children having yogurt for breakfast has steadily increased. In 2000, only 1% of children reported having yogurt for breakfast. This percentage was the same for both year groups and genders. This increased to 7% for males and 5% for females in 2012. The percentage of school children having fruit for breakfast has increased more dramatically for all ages. For males in year 8, there is an increase from 2 to 18% and in year 10 from 1 to 15%. Females in year 8 show a rise from 3 to 19% and in year 10 from 3 to 15%.

### Lunch Choices

**Figure 4:**
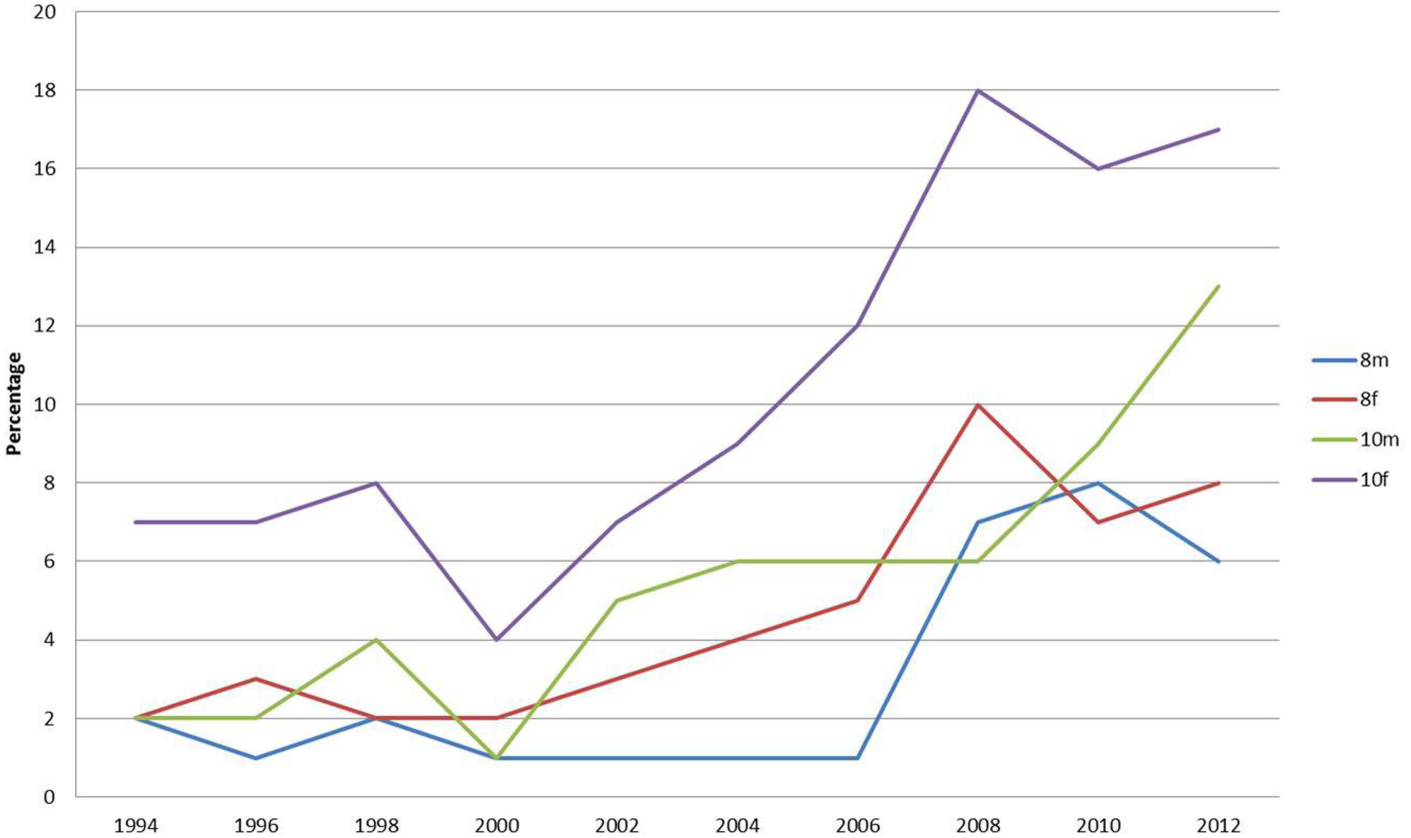
**Percentage of respondents reporting that they did not have lunch**.

The percentage of pupils reporting that they did not have lunch has increased progressively across the study period in all categories (Figure 4). However, these percentages are relatively small. Year 10 females are significantly different from all others in skipping lunch. In 2008, year 10 females were twice as likely to skip lunch as year 8 females (the next highest group). Since 2008, the percentage of year 10 males skipping lunch has more than doubled.

### Percentage Staying for School Lunch

**Figure 5:**
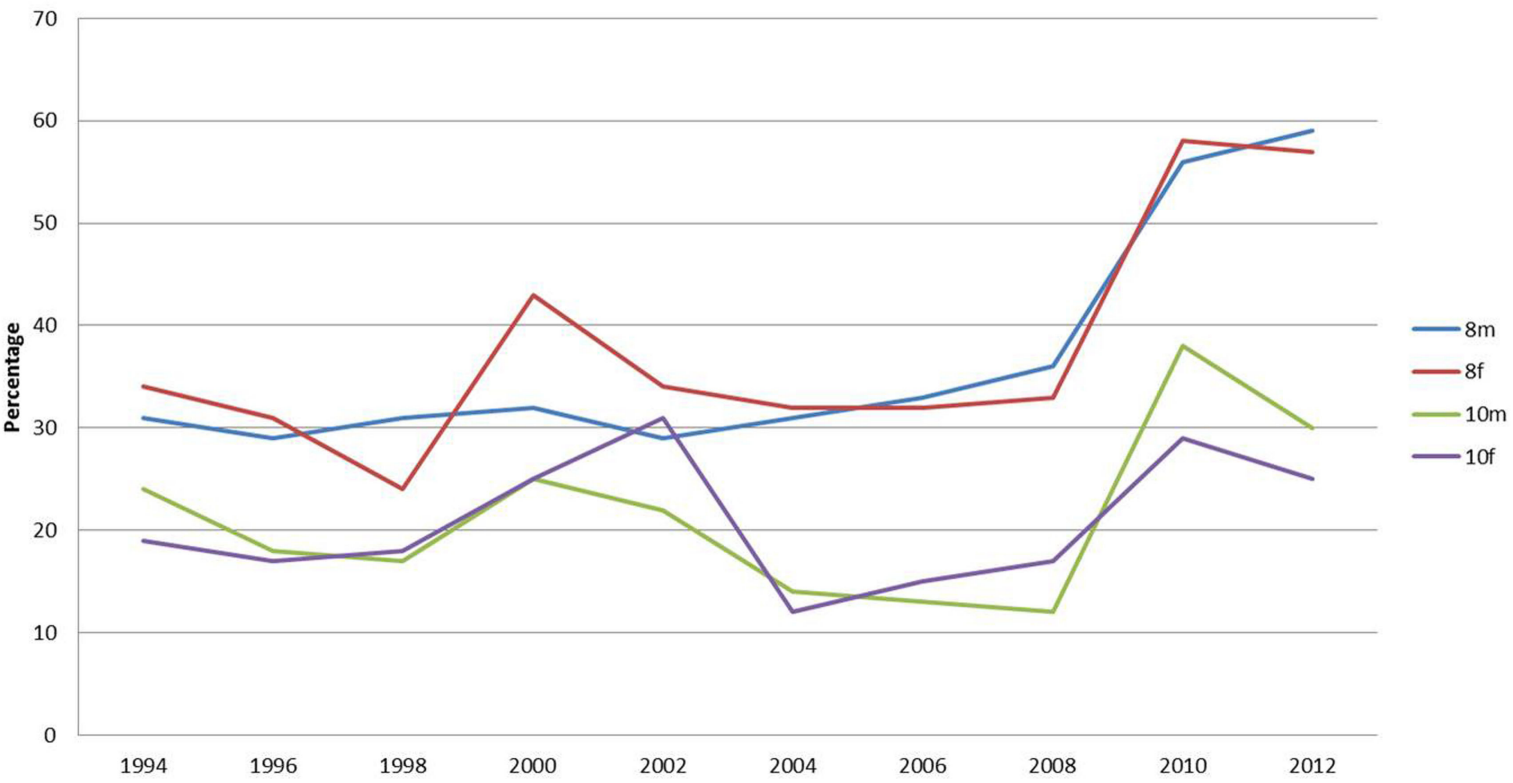
**Percentage of respondents reporting that they stayed at school for lunch**.

The percentage of school children reporting staying at school for lunch has increased (Figure 5). Year 8 school children were more likely to stay at school, than year 10 pupils. The number of males in year 10 staying for school lunch has overtaken that of their female counterparts since 2008. Previously, the situation was reversed. Over half of year 8 pupils now remain in school.

### Percentage Eating Takeaways

**Figure 6:**
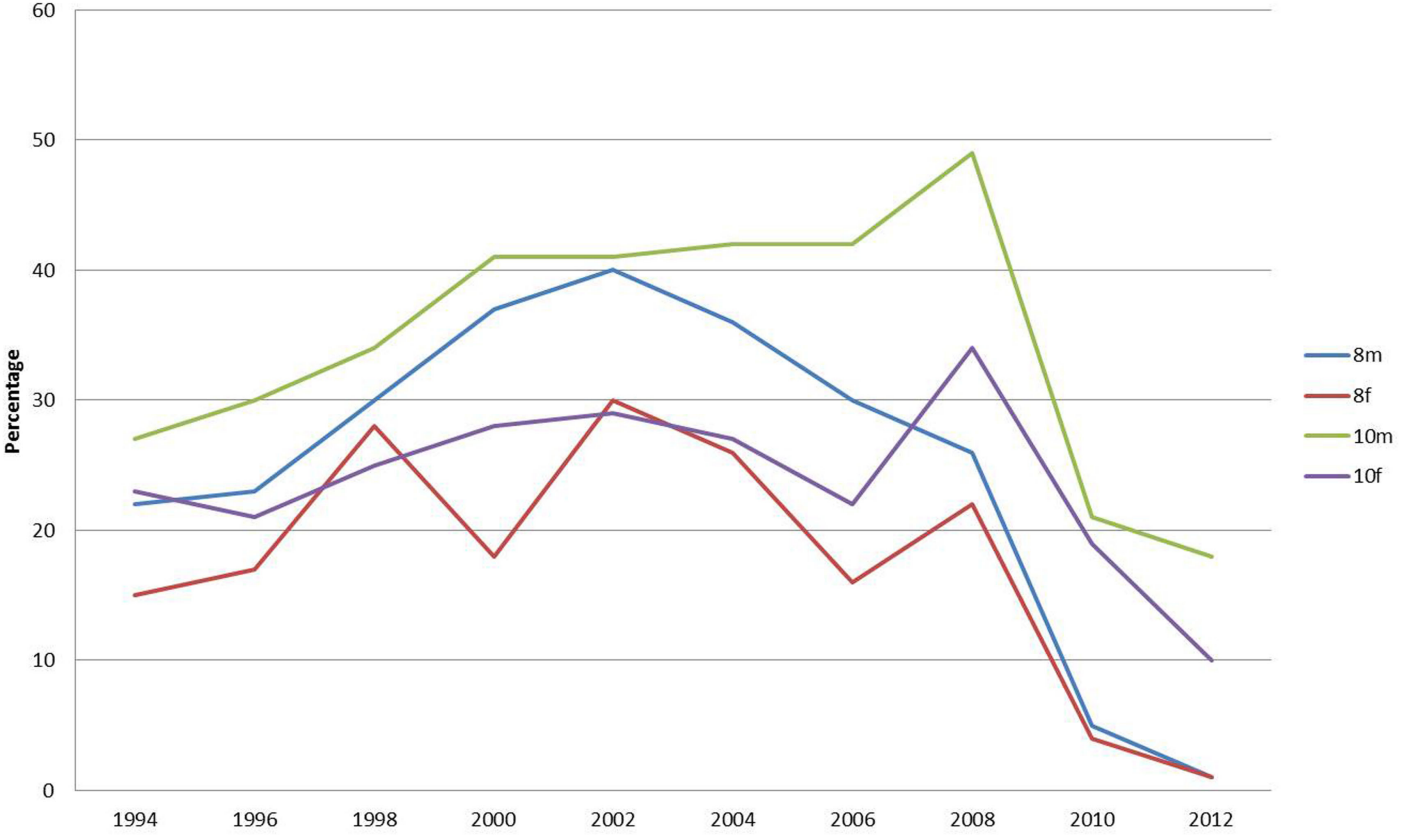
**Percentage of respondents reporting that they purchased lunch from takeaway or shop**.

Figure 6 refers to those school children buying lunch from a takeaway or shop. There has been a dramatic decrease in pupils purchasing lunch from takeaway or other retail food outlets since 2008. However, this percentage had been declining since 2002 in all groups, except year 10 males. Again, there appears to be a gender based difference with boys more likely to be using takeaways until 2006. Since then a clear age distinction between year 10 and year 8 is discernible for those eating takeaways. The number of pupils reporting buying lunch from takeaways is now below 20% for all categories, with year 8 now down to 1%.

## Discussion

It is important to note that the SHEU survey relies on self-reporting by young people. The answers provided may, therefore, represent either over or under estimation of overall consumption. However, the methodology does allow the collection of data from a relatively large sample over an extended period of time, allowing trends in behavior to be determined. It should be noted that this is not a longitudinal study, in that the same children are not followed over time, but is a time-related cross-sectional study, which permit the determination of trends. Large studies of trends are relatively uncommon within this field. The results therefore must be interpreted with these issues in mind.

### Perceptions of Weight

The percentage of school children who consider themselves overweight is relatively high with the greatest number among year 10 females. This supports the literature, although the percentage found is below the 63% reported by SHEU (29). The Sunderland survey shows that females are more likely than males to wish to lose weight. Over the survey period, two-thirds of year 10 females reported the desire to lose weight, with the peak in 2008 at 68%. This also correlates with findings from the national SHEU (29) survey. The data must be viewed in light of the fact that it is based on the school childrens’ own reported perceptions, since there are no data questions asking what the school childrens’ weight actually is. This is a problem with self-reported questionnaires of this type, as what is analyzed here is a perception and not a reality.

### Eating Behavior

Researchers have noted that large percentages of school children are coming to school hungry, not having breakfast and not having the nutrients to sustain schoolwork (30). There is evidence of school children choosing an inadequate breakfast contributing to poor food choices for the remainder of the day and risking long-term obesity (31–33) and behavior problems (34). However, this survey identified that approximately 60% of school children are having breakfast at home and over 50% are having cereals for breakfast. Recently, it has been found that there has been a dramatic increase in the percentage of school children in Sunderland having yogurt and fruit for breakfast. These findings contradict those of the Kellogg’s (21) study where it was suggested that 75–80% were not having a nutritious start to the day. Over 50% of school children in Sunderland report having a nutritious start with breakfast.

In the national survey, 14% of year 10 females have “nothing at all to eat or drink for breakfast this morning” and 20% had nothing for lunch on the previous day (35). In this Sunderland survey, 12% of year 10 females reported not eating breakfast and 17% reported having no lunch, which matched the national findings (35). The percentage of pupils staying for school lunch or bringing a packed lunch has increased consistently over the survey period, while the percentage of school children purchasing lunch from takeaway outlets has dramatically decreased, which is pleasing since health policy of limiting takeout provision is high on government agenda.

The findings of this study appear to be in direct conflict with the stereotypical view of the eating behaviors of teenage school children from provincial postindustrial cities as portrayed by the popular media in the UK. They are portrayed as overweight individuals having no proper breakfast and buying a snack (such as crisps and coke) from the corner shop on the way to school. Indeed, much has been made of the fact that children are reaching school in an undernourished state, without the energy to properly concentrate on their lessons. The findings of this study contradict this, as a large proportion in all age ranges report to having breakfast before leaving home. The percentage of school children who reported having breakfast at home has increased progressively over the period. The percentage of this group reporting eating nothing before school has also dropped progressively. Moreover, the nutritional content of breakfast items reported to be eaten appears to be nutritionally adequate. There has been a steady move away from the traditional cooked breakfast with healthy alternatives of cereals, fruit, and yogurt being consumes instead. At lunchtime, the picture painted in the media is of school children going to the local fish and chip, pizza shop, or other fast food outlet. However, this study indicated that a progressively greater number of pupils are having school lunches or taking premade lunches to eat at school. There has been a dramatic decrease in pupils purchasing lunch from takeaways or other fast food retail outlets since 2002.

It appears that school children are concerned about their weight and a high proportion report a desire to lose weight. Whether this because they are actually overweight cannot be determined, since the question was not asked. It does indicate, however, that there is a definite perception of body image. This may be due to the portrayal in the popular media of ideal body types of both males and females. There is a striking difference between the female and male responses in their desire to lose weight; consistently greater percentages of females reported this. Year 10 females are the group reporting the largest percentage wishing to lose weight. Year 10 females are significantly different from all others in skipping lunch. They are twice as likely to skip lunch as year 8 females, the next highest group. It is also of note that since 2008, the percentage of year 10 males skipping lunch has more than doubled. This may be a result of the increased media attention on ideal body proportions and male grooming. At first sight, this desire to lose weight may be good, given that the region possesses a high proportion of obesity. However, the desire of females, in particular those of year 10, to lose weight coupled with the increased tendency of this group to skip meals may be problematic. It could be that this becomes the precursor to eating regimes leading to bulimia and anorexia nervosa.

Whatever the motivation it does appear that the young people answering this survey are more weight conscious, eat more healthily and make more sensible health choices than expected from the stereotypical media presentations. Perhaps the numerous health education campaigns that have taken place nationally and more focused initiatives within Sunderland have resulted positively in a change to progressively beneficial trends in eating behavior.

## Conclusion

The findings of the Sunderland study support other national research that the majority of female school children perceive that they want to lose weight. In Sunderland, school children have access to a range of specific services to combat obesity. This includes a highly successful Lifestyle, Activity, and Food program, the Sunderland Association Football Club (SAFC) Wider Family Learning courses, a Healthy Schools Program which incorporate a proactive approach to increasing physical activity and providing school meals that meet all nutritional standards.

The Sunderland research identifies that not as many school children in Sunderland are skipping breakfast when compared to the national trend. This is pleasing considering this is a deprived area and food poverty may be perceived as being high in the area. Furthermore, significant numbers of school children in Sunderland are eating a nutritious breakfast at home. It may be that the initiatives which have taken place in schools in Sunderland have influenced the food choices of school children, which may result in the opportunity to reduce social inequalities (10).

An overall downward trend over the survey period in Sunderland of school children skipping lunch and purchasing takeaways from fast food outlets was found. Increasing numbers are staying at school to eat lunch. These are pleasing trends and match the national government policies to tackle both of these issues. This contradicts the adverse media portrayal and perception of obesity in Sunderland, including regular takeaway meal consumption and skipping meals. Public health interventions have arguably led to an efficacious behavior change among school children to bring about health improvement.

Sunderland’s has an innovative Local Authority with a clear and mature vision for tackling health inequalities and improving public health (36–38), and this aligned with a series of other initiatives within the city, including the SAFC programs aim to improve the wider social determinants of health.

Overall, the findings of the survey show that the eating behaviors and food choices of school children in Sunderland do not match up to those portrayed in the national and local press. It may be that this adverse publicity aligned with the initiatives of the LA, has fundamentally altered these behaviors and Sunderland school children are now bucking the trend of obesity.

## Author Contributions

AM and DB contributed equally to the conception, analysis and interpretation, drafting and final approval of this paper.

## Conflict of Interest Statement

The authors declare that the research was conducted in the absence of any commercial or financial relationships that could be construed as a potential conflict of interest.

## Abbreviations

SHEU, Schools and Students Health Education Unit; UK, United Kingdom; LAs, Local Authorities; SAFC, Sunderland Association Football Club.
